# Supplementary material for: Effects of Food Source Availability, Host Egg:Parasitoid Ratios, and Host Exposure Times on the Developmental Biology of Megacopta cribraria Egg Parasitoids
Source: Insects. 2023 Sep 9;14(9):755. doi: 10.3390/insects14090755 (PMC10531800; doi:10.3390/insects14090755)
Supplement: Supplementary file 1 [file insects-14-00755-s001.zip › insects-2564862-supplementary.pdf]

Research Article

Effects of food source availability, host egg: parasitoid ratios, and host exposure times on the developmental biology of *Megacopta cribraria* egg parasitoids

Sanower Warsi[1], Ana M. Chicas-Mosier[2], Rammohan R. Balusu[3], Alana L. Jacobson[1*] Henry Y. Fadamiro[4]

[1] Department of Entomology and Plant Pathology, Auburn University, Auburn, AL, USA; szw0132@auburn.edu, alj0043@auburn.edu
[2] Center for Environmentally Beneficial Catalysis, University of Kansas, Lawrence, Kansas, USA; ana.chicasmosier@ku.edu
[3] BASF Agricultural Products Group, Research Triangle, NC, USA; rammohan.balusu@basf.com
[4] Department of Entomology, Texas A&M University, College Station, TX, USA; henry.fadamiro@tamu.edu
* Correspondence: alj0043@auburn.edu Tel.: (334) 844-5011.

Supplement file

Table S1. Parametric Two-Way ANOVA to evaluate the effects of the constant supply of water and honey on several life-history traits of *Ooencyrtus nezarae* reared on eggs of *Megacopta cribraria*, throughout the parasitoid's lifespan. *N* = (10) water, (10) honey, (10) water + honey, and (10) fasting. One mated individual wasp formed each replicate.

Life history trait	Source of variation	df	F	*p*-value
Host eggs parasitized	Diet	3, 253	16.37	<0.0001
	Age	29, 253	3.15	<0.0001
	Diet and age interaction	31, 253	1.12	0.315
Emerged host nymphs	Diet	3, 253	7.30	0.0001
	Age	29, 253	1.87	0.0058
	Diet and age interaction	31, 253	1.41	0.079
Wasp offspring	Diet	3, 253	13.44	<0.0001
	Age	29, 253	3.30	<0.0001
	Diet and age interaction	31, 253	0.96	0.528
Offspring sex ratio	Diet	3, 253	7.17	0.0001
	Age	29, 253	2.40	0.0002
	Diet and age interaction	31, 253	0.66	0.917

Table S2. Parametric Two-Way ANOVA to evaluate the effects of the constant supply of water and honey on several life-history traits of *Paratelenomus saccharalis* reared on eggs of *Megacopta cribraria*, throughout the parasitoid's lifespan. *N* = (8) water, (8) honey, (8) water + honey, and (8) fasting. One mated individual wasp formed each replicate.

Life history trait	Source of variation	df	F	*p*-value

Host eggs parasitized	Diet	3, 141	2.12	0.1002
	Age	13, 141	17.80	<0.0001
	Diet and age interaction	13, 141	1.98	0.026
Emerged host nymphs	Diet	3, 141	2.34	0.0763
	Age	13, 141	10.84	<0.0001
	Diet and age interaction	13, 141	1.51	0.120
Wasp offspring	Diet	3, 141	1.91	0.1309
	Age	13, 141	16.28	<0.0001
	Diet and age interaction	13, 141	2.26	0.0101
Offspring sex ratio	Diet	3, 141	2.44	0.066
	Age	13, 141	3.47	0.0001
	Diet and age interaction	13, 141	1.27	0.235

Table S3. Results of a Kruskal–Wallis ANOVA with Dunn's post hoc test comparing the proportion of eggs parasitized, emerged *M. cribraria* nymphs, wasp offspring, and offspring sex ratio (female/total) compared between different host densities of *Ooencyrtus nezarae* [sample size (n)= 20] and *Paratelenomus saccharalis* [sample size (n)= 10]. The 'z' value represents the standardized test statistic derived from Dunn's post hoc test, which measures the difference between ranks of two groups. A higher absolute z-value indicates a greater difference between the two groups being compared.

Parasitoid species	Experiment	Host density comparison	Host eggs parasitized	Emerged *M. cribraria* nymphs	Wasp offspring	Offspring sex ratio
Ooencyrtus nezarae	**Single wasp**	21 vs. 42	z =1.84, p =0.06	z =-1.74, p =0.08	z =1.40, p = 0.16	z =-1.43, p =0.15
		21 vs. 84	z =6.09, **p < 0.001**	z =-5.71, **p < 0.001**	z =5.71, **p < 0.001**	z =-1.56, p =0.11
		21 vs. 168	z =7.87, **p < 0.001**	z =-6.68, **p < 0.001**	z =7.94, **p < 0.001**	z =-0.22, p =0.82
		42 vs. 84	z =4.25, **p < 0.001**	z =-3.96, **p < 0.001**	z =4.30, **p < 0.001**	z =-0.13, p =0.89
		42 vs. 168	z =6.03, **p < 0.001**	z =-4.93, **p < 0.001**	z =6.54, **p < 0.001**	z =1.20, p =0.22
		84 vs. 168	z =1.77, p = 0.07	z =-0.97, p =0.33	z =2.23, **p = 0.02**	z =1.34, p =0.18
			(H = 80.18, df =3, **p < 0.001)**	(H = 60.71, df =3, **p < 0.001)**	(H = 82.10, df =3, **p < 0.001)**	(H = 3.90, df =3, p =0.27)
	**Seven wasps**	21 vs. 42	z =2.02, **p =0.04**	z =-1.65, p =0.09	z =-1.45, p = 0.14	z =-1.54, p = 0.12
		21 vs. 84	z =3.24, **p =0.001**	z =-3.94, **p < 0.001**	z =-1.91, **p = 0.05**	z =-2.04, p = 0.04
		21 vs. 168	z =7.78, **p < 0.001**	z =-8.82, **p < 0.001**	z =2.39, **p = 0.01**	z =-1.39, p = 0.16
		42 vs. 84	z =1.22, p =0.22	z =-2.29, **p = 0.02**	z =-0.46, p = 0.64	z =-0.50, p = 0.61
		42 vs. 168	z =5.76, **p < 0.001**	z =-7.17, **p < 0.001**	z =3.85, **p < 0.001**	z =0.14, p = 0.88
		84 vs. 168	z =4.53, **p < 0.001**	z =-4.88, **p < 0.001**	z =4.31, **p < 0.001**	z = 0.64, p = 0.51
			(H = 65.20, df =3, **p < 0.001)**	(H = 88.36, df =3, **p < 0.001)**	(H = 22.62, df =3, **p < 0.001)**	(H = 4.60, df =3, p =0.20)
Paratelenomus saccharalis	**Single wasp**	21 vs. 42	z =0.94, p = 0.34	z = -0.96, p =0.33	z =0.53, p = 0.59	z = -0.54, p =0.58
		21 vs. 84	z =4.29, **p < 0.001**	z = -4.15, **p < 0.001**	z =3.73, **p < 0.001**	z = 1.07, p =0.28
		21 vs. 168	z =6.78, **p < 0.001**	z = -7.10, **p < 0.001**	z =6.35, **p < 0.001**	z = -0.67, p =0.49
		42 vs. 84	z =3.34, **p < 0.001**	z = -3.17, **p = 0.001**	z =3.20, **p = 0.001**	z = 1.62, p =0.10
		42 vs. 168	z =5.84, **p < 0.001**	z = -6.14, **p < 0.001**	z =5.82, **p < 0.001**	z = -0.14, p =0.88
		84 vs. 168	z =2.58, **p = 0.01**	z = -3.04, **p = 0.002**	z =2.69, **p = 0.007**	z = -1.74, p =0.08
			(H = 58.24, df =3, **p < 0.001)**	(H = 62.37, df =3, **p < 0.001)**	(H = 52.60, df =3, **p < 0.001)**	(H = 3.83, df =3, p = 0.28)
		21 vs. 42	z =-0.48, p = 0.62	z = -1.63, p =0.10	z = -2.12, **p =0.03**	z = -3.59, **p <0.001**

	**Seven wasps**	21 vs. 84	*z* =1.01, *p* = 0.30	*z* = -2.48, ***p* =0.01**	*z* = -0.85, *p* =0.39	*z* = -3.13, ***p* =0.002**
		21 vs. 168	*z* =2.85, ***p* = 0.004**	*z* = -4.97, ***p* < 0.001**	*z* =1.15, *p* = 0.24	*z* = -4.65, ***p* <0.001**
		42 vs. 84	*z* =1.50, *p* = 0.13	*z* = -0.84, *p* =0.39	*z* =1.27, *p* = 0.20	*z* =0.45, *p* = 0.64
		42 vs. 168	*z* =3.34, ***p* < 0.001**	*z* = -3.33, ***p* < 0.001**	*z* =3.28, ***p* = 0.001**	*z* = -1.06, *p* =0.28
		84 vs. 168	*z* =1.84, *p* = 0.06	*z* = -2.49, ***p* =0.01**	*z* =2.01, ***p* = 0.04**	*z* = -1.52, *p* =0.12
			(*H* = 13.14, df =3, ***p* = 0.004**)	(*H* = 25.84, df =3, ***p* < 0.001**)	(*H* = 11.51, df =3, ***p* = 0.009**)	(*H* = 24.01, df =3, ***p* < 0.001**)

Significant values ($p \leq 0.05$) are in bold font.

Table S4. Results of a Kruskal–Wallis ANOVA with Dunn's post hoc test comparing the proportion of *Megacopta cribraria* eggs parasitized, emerged *M. cribraria* nymphs, wasp offspring, and offspring sex ratio (female/total) among host exposure times (days) for *Ooencyrtus nezarae* [sample size (n)= 20] and *Paratelenomus saccharalis* [sample size (n)= 10]. The 'z' value represents the test statistic from the Dunn's post hoc test, indicating the strength and direction of differences between comparisons.

Parasitoid species	Experiment	Host exposure time comparison	Host eggs parasitized	Emerged *M. cribraria* nymphs	Wasp offspring	Offspring sex ratio
Ooencyrtus nezarae	Single wasp	1 vs. 3	z = -6.19, $\mathbf{p}$ **< 0.001**	z =4.81, $\mathbf{p}$ **< 0.001**	z =-5.88, $\mathbf{p}$ **< 0.001**	z =-2.65, $\mathbf{p}$ **=0.008**
		1 vs. 5	z = -7.71, $\mathbf{p}$ **< 0.001**	z =7.63, $\mathbf{p}$ **< 0.001**	z =-7.25, $\mathbf{p}$ **< 0.001**	z =-0.72, p =0.46
		3 vs. 5	z = -1.51, p = 0.12	z =2.81, $\mathbf{p}$ **= 0.005**	z =-1.36, p =0.17	z =1.92, $\mathbf{p}$ **=0.05**
			(H=66.76, df =2, $\mathbf{p}$ **< 0.001**)	(H=59.59, df =2, $\mathbf{p}$ **< 0.001**)	(H=59.48, df =2, $\mathbf{p}$ **< 0.001**)	(H=7.52, df =2, $\mathbf{p}$ **= 0.02**)
	Seven wasps	1 vs. 3	z = -4.15, $\mathbf{p}$ **< 0.001**	z =4.02, $\mathbf{p}$ **< 0.001**	z =-1.29, p =0.19	z =-0.42, p =0.67
		1 vs. 5	z = -5.78, $\mathbf{p}$ **< 0.001**	z =5.38, $\mathbf{p}$ **< 0.001**	z = 0.80, p =0.42	z = 0.38, p =0.70
		3 vs. 5	z = -1.62, p = 0.10	z =1.35, p = 0.17	z =2.09, p =0.03	z = 0.80, p =0.42
			(H=36.63, df =2, $\mathbf{p}$ **< 0.001**)	(H=31.32, df =2, $\mathbf{p}$ **< 0.001**)	(H=4.46, df =2, p = 0.10)	(H=0.64, df =2, p = 0.72)
Paratelenomus saccharalis	Single wasp	1 vs. 3	z = -0.95, p = 0.34	z = 1.75, p = 0.07	z = -1.08, p = 0.28	z = 2.60, $\mathbf{p}$ **= 0.009**
		1 vs. 5	z = -1.17, p = 0.24	z = 1.20, p = 0.22	z = -0.87, p = 0.38	z = 0.39, p = 0.69
		3 vs. 5	z = -0.21, p = 0.82	z = -0.55, p = 0.58	z = 0.20, p = 0.83	z = -2.21, $\mathbf{p}$ **= 0.02**
			(H = 1.55, df =2, p = 0.46)	(H = 3.23, df =2, p = 0.19)	(H = 1.32, df =2, p = 0.51)	(H = 7.89, df =2, $\mathbf{p}$ **= 0.01**)
	Seven wasps	1 vs. 3	z = -0.97, p = 0.32	z = 0.53, p = 0.59	z = -1.07, p = 0.28	z = -0.27, p = 0.78
		1 vs. 5	z = 1.16, p = 0.24	z = -1.94, $\mathbf{p}$ **= 0.05**	z = 1.02, p = 0.30	z = 0.85, p = 0.39
		3 vs. 5	z = 2.14, p = 0.03	z = -2.47, $\mathbf{p}$ **= 0.01**	z = 2.10, p = 0.03	z = 1.12, p = 0.25
			(H = 4.59, df =2, p = 0.10)	(H = 6.79, df =2, $\mathbf{p}$ **= 0.03**)	(H = 4.41, df =2, p = 0.11)	(H = 1.38, df =2, p = 0.49)

Significant values ($p \leq 0.05$) are in bold font.
